# Investigation of the Binary Nitrides YN, LaN and LuN by Solid-State NMR Spectroscopy

**DOI:** 10.3390/molecules29235572

**Published:** 2024-11-25

**Authors:** Jennifer Steinadler, Georg Krach, Wolfgang Schnick, Thomas Bräuniger

**Affiliations:** Department of Chemistry, University of Munich (LMU), Butenandtstr. 5-13, 81377 Munich, Germany

**Keywords:** ^89^Y^14^N, ^139^La^14^N, Lu^14^N, binary nitrides, MAS-NMR, isotropic chemical shifts

## Abstract

Based on their various and outstanding properties, binary nitrides are used as (synthesis) materials in industry and research. Hence, their comprehensive characterization by analytical methods is of particular interest. Since Nuclear Magnetic Resonance (NMR) spectroscopy is very sensitive to the symmetry of the electronic density distribution, it is a suitable tool for the investigation of rock-salt structure types and, especially, for those with known stoichiometry issues. Here, we report on magic-angle spinning NMR spectra of the nuclides ^89^Y (I=12), ^139^La (I=72) and ^14^N (I=1) in polycrystalline samples of YN, LaN and LuN. Due to the high symmetry of their crystal structures, the spectra of all nuclides do not exhibit anisotropic effects of significant magnitude. The resulting isotropic chemical shift values are $\delta_{iso}$(^89^Y) = 516 ppm for YN, $\delta_{iso}$(^139^La) =1294 ppm for LaN, and $\delta_{iso}$(^14^N) = 457 ppm (YN), 788 ppm (LaN) and 384 ppm (LuN). The newly determined $\delta_{iso}$(^14^N) values for these three binary nitrides fit well into the previously reported linear correlation between nitrogen distance to the nearest cation and isotropic chemical shift, leading to a better correlation coefficient and reduced error margins for the fit parameters.

## 1. Introduction

Binary nitrides are widely applied in industry and daily-life products covering a broad field of various applications. For example, the hexagonal form of boron nitride is an excellent lubricant in a wide temperature range [1]. AlN possesses both high thermal conductivity and piezoelectric properties and can be found, as wide-bandgap material combined with gallium or indium to III-nitride semiconductors, in light-emitting diodes [2,3]. Furthermore, 3d-metal nitrides like TiN are used in microelectronics [4], and VN with its large electrochemical potential window is of interest for supercapacitors [5]. Also, yttrium and lanthanum nitride might be of use in electronic and/or optoelectronic applications [6]. However, for cations beyond the main-group elements, as for example transition metals, the synthesis of compounds with defined stoichiometry becomes a challenging task. The deviation from the 1:1 ratio leads to nitrogen vacancies that might actually contribute to the stabilisation of the rock-salt structure types [7]. This defect formation also produces free charge carriers and strongly affects the material properties, which has been investigated mainly theoretically by means of quantum mechanical calculations [8,9,10,11]. There have been efforts to realize samples with ideal 1:1 stoichiometry, using optimized conditions or special approaches, including laser ablation [12], ion beam sputtered coatings [13] or supercritical nitrogen fluids in laser-heated diamond anvil cells [14]. There have been attempts to quantify the nitrogen content in the products by different analytical methods like the ones of Dumas [15] or Khjeldal [16], with synthesis of single crystals also reported [17,18]. Despite all these efforts, very few clear and reliable synthesis instructions can be found in the literature, especially for bulk samples. But while it is desirable to obtain stoichiometric products for the investigation of nanoparticles, thin films, powder samples and single crystals, defects and vacancies can actually be advantageous for applications like the catalytic effects of surface nitrogen-vacancies in lanthanum nitride to enable N_2_ activation for ammonia synthesis [19].

Regardless of the desired stoichiometry of the product, nuclear magnetic resonance (NMR) spectroscopy is a sensitive and at the same time non-destructive technique which is helpful for the analysis of binary nitrides. Since NMR directly probes the local electronic environment of a nucleus, valuable additional information complementing X-ray analysis may be obtained. Due to its sensitivity to small deviations in the local geometric and electronic environment of the nucleus of interest, it is a suitable method to detect even small amounts of vacancies [20,21] and impurities [22,23]. While NMR data are already available on several binary nitrides [24,25,26,27,28], the current work will extend the series to include three more representatives, namely with the 4d- and 5d-metals yttrium and lanthanum as well as the rare-earth element lutetium as cations. These elements were deliberately chosen so that in theory, only closed-shell states should be present for ideal 1:1 compositions. For other samples, i.e., TiN and VN, the valence electrons of the cations lead to the presence of paramagnetic properties [29,30], which made it necessary to dilute them in the NMR rotor for magic angle spinning (MAS) experiments [28,31]. For the binary nitrides investigated in this work, we report NMR spectra of the nuclides ^89^Y and ^139^La, and those of ^14^N. In the following, their evaluation as well as the linear correlation between the obtained isotropic chemical shift values for ^14^N and the shortest distances of the nitrogen atoms to the next cation in the respective structures will be described in detail.

## 2. Results and Discussion

The investigated compounds can be denoted as ^89^Y^14^N, ^139^La^14^N and Lu^14^N in good approximation due to the natural abundance of these nuclides, i.e., 100% for ^89^Y, 99.91% for ^139^La and 99.64% for ^14^N, indicating the most suitable choices for NMR experiments. In a strong external magnetic field, the various interactions each nucleus experiences at its position in the crystal structure can be considered as perturbations to the Zeeman transition energies. The influence of these interactions on the resonance frequencies may be expressed by the following equation:

(1) $$\nu =\nu_{0}+\nu_{CS}+\nu_{IS}+\nu_{k}^{\left(1\right)}+\nu_{k^{2}}^{\left(2\right)}+\ldots$$

For nuclei with spin I=12, like the ^89^Y isotope in this work, the respective resonance frequency ν is predominantly determined by their Larmor frequency $\nu_{0}$, which is slightly modified by the chemical shift $\nu_{CS}$ and dipolar couplings $\nu_{IS}$. For quadrupolar nuclei, the interaction of their non-spherical charge distributions with the electric field gradient (EFG) of the surrounding must be taken into account, usually by means of perturbation theory to first and second order [32]. These contributions are expressed by $\nu_{k}^{\left(1\right)}$ and $\nu_{k^{2}}^{\left(2\right)}$, respectively, in the above equation. Here, the parameter *k* denotes the relevant transition, being connected to the familiar magnetic quantum number *m* for the transition |m〉→|m±1〉 by [33,34]:

(2) $$k=m\pm \frac{1}{2}$$

For purpose of quantifying the quadrupolar interaction, the coupling constant χ (or $C_{Q}$) in units of [Hz] and the dimensionless asymmetry parameter $\eta_{Q}$ are commonly used. From the definition of χ (given below) it may be seen that this quantity scales directly with the quadrupole moment *Q* [35] of the respective nucleus and the largest eigenvalue $V_{33}$ of the EFG tensor, whereas $\eta_{Q}$ is defined as a ratio of **V**-tensor elements (with $|V_{11}\left|\leq \right|V_{22}\left|\leq \right|V_{33}|$):

(3) $$\chi =\frac{eQ}{h}V_{33}\;\eta_{Q}=\frac{V_{11}-V_{22}}{V_{33}}$$

Considering the cubic symmetry of the investigated nitrides which all crystallize in space group $Fm\bar{3}m$ (no. 225), both cations and anions are located at the centers of regular octahedra of the respective counter ion. Furthermore, the criterion of an highly isotropic environment is fulfilled not only in this first coordination sphere but also in the more distant ones. Hence, in the ideal rock-salt structure type, the quadrupolar coupling constants χ for nuclides with spin I>12 are expected to be zero. Even for small structural distortions, which would lead to non-zero χ’s, the asymmetry parameter $\eta_{Q}$ should be zero due to the occupation of Wyckoff positions with three-fold rotational symmetry by all atoms inducing cylindrical symmetry.

Regarding dipolar couplings, their effect on an isolated, heteronuclear spin pair scales directly with the gyromagnetic ratios γ of the two spins *I* and *S* and inversely with their cubed distance $r_{IS}$ to each other. As a measure for the interaction strength, the so-called dipolar coupling constant $b_{IS}=-\left(\mu_{0}\gamma_{I}\gamma_{S}\hbar \right)/\left(8\pi^{2}r_{IS}^{3}\right)$ is commonly used. Calculating $b_{IS}$ for the possible spin combinations in the three investigated binary nitrides, the resulting absolute values do not exceed 73.5 Hz, which is the largest value found for the heteronuclear case of ^175^Lu–^14^N. Hence, under MAS conditions with a spinning rate of 10 kHz, such small contributions should be completely averaged out, and therefore show no net effect on our spectra.

For LuN, the isotope ^175^Lu with its natural abundance of 97.41% appears to be also a suitable candidate for NMR experiments. However, compared to the already large quadrupolar moment Q=20 fm^2^ of ^139^La [36], this quantity is more than 15 times larger for ^175^Lu. Being one of the largest known for non-radioactive isotopes it has a value of Q=349 fm^2^ [37]. Therefore, vacancies or impurities in the crystal structure of LuN are expected to have an enormous influence on the spectral appearance of the resonance line of ^175^Lu. Due to the more ionic bonding character between lutetium and nitrogen, the formation of such defects is more probable than for the heavier homologues of binary Lu pnictides which have been already investigated by means of NMR spectroscopy [38]. Under these unfavorable conditions we did not succeed in obtaining a spectrum of ^175^Lu.

### 2.1. NMR of Spin-1: *^14^*N

For a spin I=1 nucleus like ^14^N, there exist two single-quantum transitions with $k=\pm \frac{1}{2}$, see Equation (2). The two significant contributions influencing the position and the shape of the resonance line are the chemical shift and the quadrupolar interaction. The effects of the latter on our samples are described by:

(4) $$\nu_{k}^{\left(1\right)}\overset{I=1}{=}k\cdot \frac{3\chi }{2}\cdot \frac{3\cos^{2}\beta -1}{2}$$

**Figure 1:**
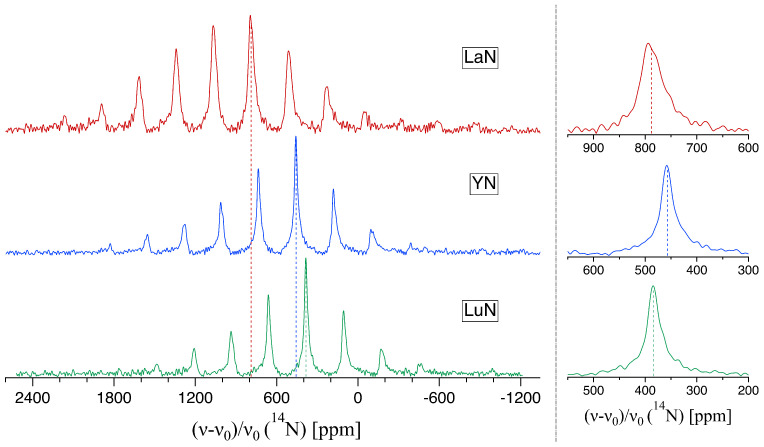
^14^N-NMR MAS spectra at 10 kHz spinning speed for polycrystalline samples of LaN, YN and LuN with the vertical lines indicating the respective isotropic chemical shift.

For the rock-salt structure the quadrupolar interaction should vanish completely, but the ^14^N spectra of all the nitrides investigated here (see Figure 1) show a substantial pattern of spinning sidebands (SSBs). These SSBs are caused by the presence of anisotropic interactions, whose source can be quadrupolar coupling effects but might as well be chemical shift anisotropies (CSA). The breakdown of the ideal symmetry of the surroundings is most likely caused by nitrogen vacancies or oxygen impurities in the crystal structure. As outlined in the Introduction, achieving a perfect 1:1 stoichiometry and phase purity is a very challenging task for this compound class. The distribution of vacancies and/or impurities throughout the crystal structure results in additional line broadening, as can be seen especially in the spectrum of La^14^N. While these effects are smaller in the case of Y^14^N and Lu^14^N, a certain distribution of NMR parameters is also present in their corresponding spectra. This is indicated by the characteristic distribution tailing of each resonance line in the spectrum towards lower frequencies [39]. Overall, however, the envelopes of the SSB patterns do not show the characteristic lineshape with two pronounced maxima (belonging to the two $k=\pm \frac{1}{2}$ transitions) as it would be expected for static ^14^N powder spectra under the influence of first-order quadrupolar interactions (FOQI) [40]. This effect persists experimentally even for relatively small quadrupolar coupling constants of about 140 kHz at an MAS rate of 12 kHz, as has been observed for *h*-BN [24]. Consequently, possible FOQI effects have to be significantly smaller for the compounds investigated here, and second-order effects will be practically absent. Under the assumption that the observed SSB patterns are caused chiefly by the quadrupolar interaction, the ^14^N spectra were simulated using the Simpson [41] package (see Supplementary Material). Comparing the results with the obtained spectra in Figure 1, an upper limit for χ of about 43 kHz was determined for LaN, since for larger values of χ, the isotropic peak is not the one with the highest intensity anymore. For LuN and, especially, for YN with a significantly less intense sideband pattern, χ has to be even smaller.

The position of the resonances of highest intensity in the spectra can be identified with the isotropic chemical shift of the ^14^N isotope, with the assignment being verified by additional measurements with a spinning rate of 8 kHz. This leads to $\delta_{iso}$ values of 788 ppm for LaN, 457 ppm for YN and 384 ppm for LuN.

### 2.2. *^14^*N Chemical Shift Relation to Crystal Structure

Correlation effects between NMR interaction parameters and crystal structure are of particular interest as a possible means to aid structure elucidation by NMR spectroscopy. One such possibility is to correlate the values of the isotropic chemical shift with the average distance to neighbouring atoms in the first coordination sphere for the observed nuclide [42,43,44]. To avoid the fraught debate about the constituting atoms of a coordination polyhedron (i.e., which atoms in the structure are actually considered to be neighbours), it has been suggested to just use the distance to the nearest atom, which is also an indirect measure of the packing density of the structure. This approach has been successfully applied to examples with ^207^${\mathrm{PbO}}_{x}$, ^23^${\mathrm{NaO}}_{x}$, ^27^${\mathrm{AlO}}_{x}$ and ^43^${\mathrm{CaO}}_{x}$ polyhedra, regardless of the compound class [45,46] and has been carried over to the class of binary nitrides with good results [28].

**Figure 2:**
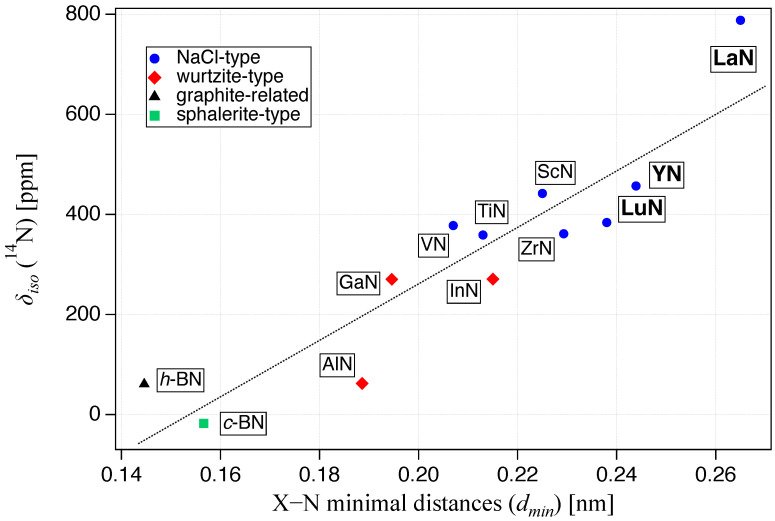
Isotropic chemical shift values $\delta_{iso}$, plotted against the shortest existing X–N distance in the respective crystal structures. Actual data and references are listed in Table 1. The dotted line represents the fit line according to $\delta_{iso}{(}^{14}N)=a+b\cdot d_{min}$ with the obtained fit parameters being a=−867±180 and b=5642±900, with a Pearson correlation coefficient *r* of 0.91.

In this work, three new entries are added to this correlation (see Table 1). In fact, to look for the ^14^N signals of the new nitrides we successfully used the previously published correlation [28] to estimate the required spectral range.With Y being the first 4d metal and, La and Lu situated even in the next period of the periodic table, the ^14^N chemical shift of their binary nitrides extended the existing graph to larger atomic distances. In comparison to the previously published plot [28], the new values not only put this linear correlation on a broader base of data points, but they also reduce the errors of the fit parameters, while slightly improving the Pearson correlation coefficient. In Figure 2, all values of $\delta_{iso}$(^14^N), as measured by us or obtained from the literature, are plotted against the shortest distance to the next cation. The corresponding crystal structures were taken from the Inorganic Crystal Structure Database (ICSD, provided by FIZ Karlsruhe GmbH), choosing the one closest to the average of frequently published values.

### 2.3. NMR of Cations

In the case of ^139^La, primarily the quadrupolar interaction as well as the chemical shift have to be considered. The spectra of nuclei with spin I=72 consist of a central transition (CT) with k=0, symmetrically flanked by satellite transitions (ST) with k=±1, ±2, ±3. While the position of the CT is independent of first-order effects, the spacing of the STs is directly proportional to χ as it can be seen from the following equation (for η=0):

(5) νk(1)=I=72k·χ14·3cos2β−12

**Figure 3:**
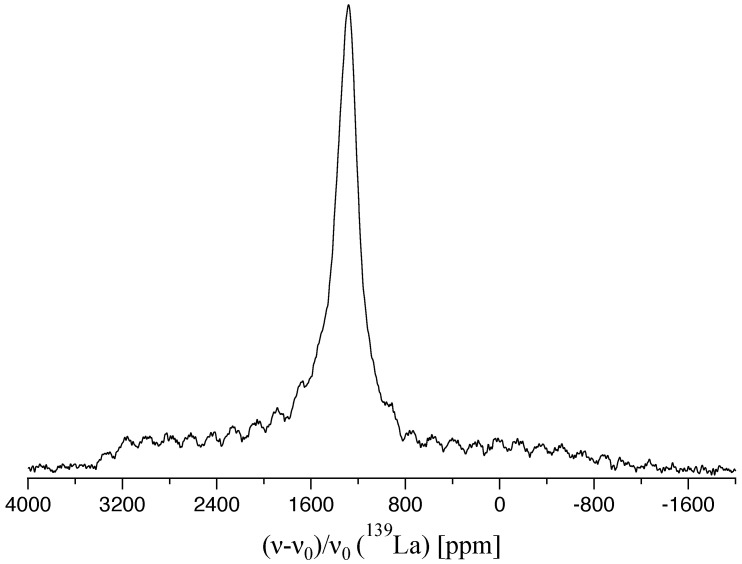
^139^La-NMR MAS spectrum at 10 kHz spinning rate for the polycrystalline LaN sample with the center band located at 1294 ppm.

Figure 3 shows the ^139^La spectrum of the LaN sample. Due to the distribution of vacancies and/or impurities now already occurring in the first coordination sphere of the investigated nucleus, the obtained resonance line suffers from severe line broadening and small spinning sidebands are present. This SSB pattern is again caused by residual anisotropic interactions. Nevertheless, the intense central transition is visible as a single, symmetric resonance indicating the absence of substantial second-order effects. Neglecting CSA and only assuming first-order effects, a maximum value for the qudrupolar coupling constant can be estimated from the width of the SSB pattern. Its outermost limits belonging to the STs with k=±3 span a range of approximately 300 kHz. For an angle of β=0 a value for $\chi^{max}=\left(150\;\mathrm{kHz}\cdot 14\right)/3=700$ kHz can be calculated as an upper limit from Equation (5). For a quadrupolar coupling constant of this magnitude, the corresponding SSB pattern calculated with the Simpson program for a single crystallographic site was of higher intensity and the shape of the envelope deviated from the measured one (see Supplementary Material). This reflects the fact that a substantial part of the experimentally observed broadening is due to the presence of a distribution. However, even for the overestimated value of χ=700 kHz the second-order quadrupolar-induced shift (QIS) on the signal position of the CT would be just of about 0.25 ppm [61]:

(6) νQIS(2)=I=72−1392·χ2ν0

Hence, it may be assumed that the center band is mainly affected by the chemical shift and its position at 1294 ppm can be identified in good approximation as $\delta_{iso}{(}^{139}$La) in the LaN structure.

**Figure 4:**
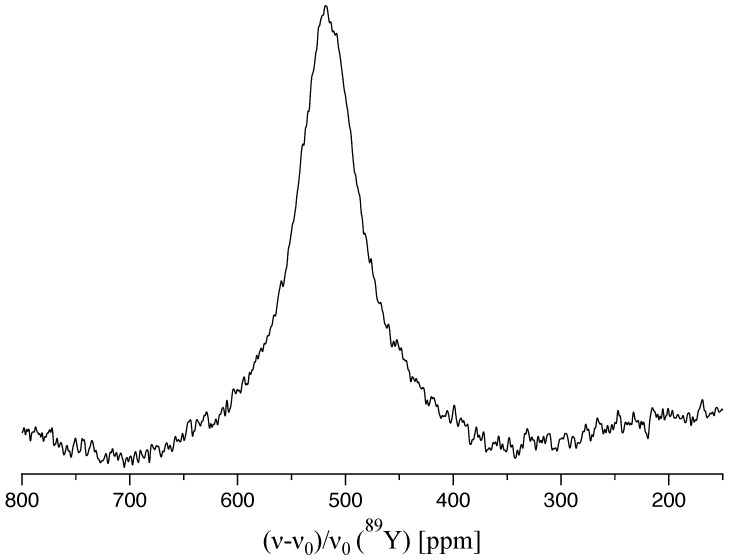
^89^Y-NMR MAS spectrum at 10 kHz spinning rate for the polycrystalline YN sample with the broad resonance line located at about 516 ppm.

Yttrium offers the advantage of being a mononuclidic element. Unfortunately, NMR measurements of this isotope are complicated by its very low gyromagnetic ratio resulting in a degraded sensitivity. In addition, long relaxation times are often present for yttrium in inorganic compounds, for example about 4 h for Y_2_O_3_ or even 6.5 h for Y_2_O_2_S [62]. Therefore, only a moderate amount of yttrium-containing compounds has been studied by means of ^89^Y NMR spectroscopy so far. Due to its spin I=12, quadrupolar coupling effects for the isotope ^89^Y are not existent and the signal position is only affected by the chemical shift. However, with its 36 electrons, Y^3+^ reacts sensitively to changes in the surrounding electron density distribution and thus, it spans a relatively wide range of chemical shift values. In addition, defects like vacancies and impurities enlarge the CSA significantly. In our case, this leads, even under MAS conditions, to a broad signal at about 516 ppm (see Figure 4). Nevertheless, the signal position is shifted to higher frequencies compared to the ones of yttrium oxide [63] as is expected for a nucleus in octahedral nitrogen coordination instead of oxygen.

## 3. Materials and Methods

The three investigated binary nitrides were synthesized by direct nitridation of metal shavings in a high-temperature approach using a radio-frequency furnace (detailed synthesis conditions as well as corresponding Rietveld analyses of the products can be found in the Supplementary Materials).

All shown NMR spectra were measured at a MAS frequency of 10 kHz and were, except for ^89^Y, carried out on a Avance-III 500 WB spectrometer (Bruker, Karlsruhe, Germany) at LMU Munich with the polycrystalline samples filled into a 4 mm rotor. Single-pulse experiments were used for the spectra of the cations while for ^14^N a spin-echo pulse-sequence was applied to minimize baseline roll and background effects [64]. The respective Larmor frequencies are $\nu_{0}{(}^{139}\mathrm{La})=70.647$ MHz and $\nu_{0}{(}^{14}N)=36.141$ MHz. Further experimental details are summarized in Table 2. Yttrium measurements were done on a Neo 600 WB instrument (Bruker, Karlsruhe, Germany) at MPI-FKF Stuttgart, at $\nu_{0}{(}^{89}Y)=29.408$ MHz. The shown spectra were all processed in magnitude (non-phase sensitive) mode and without any baseline correction. The ^14^N spectra were referenced against solid NH_4_Cl, the other nuclides against the secondary reference of the ^1^H resonance of 1% Si(CH_3_)_4_ in CDCl_3_.

## 4. Conclusions

Samples of the binary nitrides YN, LaN and LuN were synthesised and the nuclides ^89^Y, ^139^La and ^14^N investigated by means of MAS NMR spectroscopy. For the isotopes of yttrium and lanthanum, these results expand the comparatively scarce knowledge on NMR data of nitrogen-containing compounds. The spectra presented here also provide reference values for future NMR investigations especially for multinary nitride systems containing those rare-earth elements. In theory, quadrupolar coupling effects or chemical shift anisotropies should be completely absent in those cubic, rock-salt type structures leading to narrow, symmetric lineshapes. In practice, the appearance of the spectra shows the influence of nitrogen vacancies and/or oxygen impurities, which are known difficulties in the synthesis of these samples. However, these defects actually demonstrate the value of NMR as an analytical method: As long as their concentration is not high enough to affect the lattice parameters or even the atomic arrangement itself, their presence is hardly or, at least, very difficult to detect by means of PXRD experiments. This was confirmed by our experiments since the exact NMR signal positions and lineshapes vary slightly according to minor changes in the synthesis conditions, even for phase pure samples according to PXRD. The structural distortions and the distribution of NMR parameters are reflected in broad lineshapes or in a characteristic tailing of the resonance signals, respectively. This is yet another demonstration of the well-known fact that solid-state NMR of quadrupolar nuclei is very sensitive to deviations from perfect symmetry [65]. Nowadays, NMR investigations are frequently augmented by density functional theory (DFT) calculations. However, applying DFT methods to structures with imperfections is a challenging task. Commonly used program packages do not allow the straightforward calculation of the electron density distribution in combination with statistically distributed defects. This difficult and complex approach is still a topic of current research among DFT specialists [66,67,68]. Nevertheless, the results of the present work provide initial data points for possible synthesis optimization or further studies which might then be appropriately supplemented by quantum chemical calculations. Moreover, the respective ^14^N isotropic chemical shift values determined for YN, LaN and LuN fitted nicely into the previously suggested correlation of nitrogen distance to the nearest cation in each structure.

## Figures and Tables

**Table 1:** ^14^N isotropic chemical shifts, referenced against solid NH_4_Cl and listed in ascending order, for the investigated and selected binary nitrides.

Compound	Structure Type	Crystallogr. Data Ref.	$\delta_{\mathrm{iso}}$ /ppm	NMR Ref.
*c*-BN	sphalerite	[47]	−17.6	[24]
*h*-BN	graphite-related	[48]	61.2	[24]
AlN *	wurtzite	[49]	62.4	[27]
GaN *	wurtzite	[50]	270.4	[26]
InN *	wurtzite	[51]	270.9	[26]
TiN	rock-salt	[52]	359	[25]
ZrN *	rock-salt	[53]	361.4	[26]
VN	rock-salt	[54]	378	[28]
LuN	rock-salt	[55]	384	this work
ScN	rock-salt	[56]	442	[28]
YN	rock-salt	[57]	457	this work
LaN	rock-salt	[58]	788	this work

* Values for $\delta_{iso}$ re-referenced against NH_4_^+^ shifted by −342.2 ppm relative to liquid nitromethane [59] and by −355 ppm to a NO_3_^−^-solution [60].

**Table 2:** Selected measurement parameters for the shown NMR spectra with their respective recycle delays (d1), the accumulated number of scans (ns), the applied line-broadening (lb) factor and the resulting width of the isotropic peak at half the maximum height (fwhm).

	^89^YN	^139^LaN	Y^14^N	La^14^N	Lu^14^N
d1/s	240	1	180	15	240
ns	512	1000	960	6256	320
lb/Hz	50	500	0	0	0
fwhm/kHz	2.2	15.5	1.2	2.0	1.2

## Data Availability

Data is contained within the article and Supplementary Materials.

## Supplementary Materials

The following supporting information can be downloaded at: https://www.mdpi.com/article/10.3390/molecules29235572/s1, Table S1: Crystallographic data of YN, LaN and LuN from Rietveld refinement. Figures S1–S3: Rietveld refinement plots of the three synthesized binary nitrides. Figures S4 and S5: Simulations for ^14^N and ^139^La NMR spectra. The authors have cited additional references within the Supporting Materials [69,70,71,72,73,74,75].
