# Supplementary material for: Investigation of the Binary Nitrides YN, LaN and LuN by Solid-State NMR Spectroscopy
Source: Molecules. 2024 Nov 25;29(23):5572. doi: 10.3390/molecules29235572 (PMC11643925; doi:10.3390/molecules29235572)
Supplement: Supplementary file 1 [file molecules-29-05572-s001.zip › molecules-3304379-supplementary.pdf]

Supporting Information
Investigation of the Binary Nitrides YN, LaN and LuN by Solid-State NMR Spectroscopy

Jennifer Steinadler[1], Georg Krach[1],
Wolfgang Schnick[1] and Thomas Bräuniger[1,*]

[1] Department of Chemistry, University of Munich (LMU), Butenandtstraße 5–13, 81377 Munich, Germany

[*] Correspondence: Thomas.Braeuniger@cup.lmu.de; Tel.: +49-89-2180-77433

1 High-temperature synthesis of binary nitrides

The binary nitrides were synthesized in a modified route according to *Wachter* in a high-temperature approach, starting from the shavings or powder of the respective rare earth element (smart elements, 99.99 %) under N_2 atmosphere [1]. To prevent oxidation by oxygen or water, the starting materials were handled in an Ar-filled glovebox (Unilab, MBraun, Garching; O_2, $H_2O < 1$ ppm). The metal powder or shavings were filled in a tungsten crucible which was placed in the middle of the induction coil of a radiofrequency furnace (8TIG 10/100; Hüttinger Elektronik Freiburg, Germany) attached to a Schlenk line. More information about the experimental setup can be found in the literature [2]. The crucible was heated to the target temperature within 30 min, held at this temperature for 10 h, cooled down to 500 °C within 60 min, followed by switching off the furnace and natural cooling. For LaN the target temperature was set to 1400 °C, for YN to 1600 °C and for LuN to 1900 °C, respectively. Phase purity was confirmed for all samples by means of powder X-ray diffraction via a Rietveld refinement [3]. Only in the case of yttrium nitride a minor side phase of Y_2O_3 was observable. The N_2 gas was purified from possible impurities such as H_2O, CO_2 and O_2 by passing it through NaOH, orange gel, molecular sieve, P_4O_{10} and BTS catalyst [4].

2 Powder X-ray diffraction (PXRD) and Rietveld refinement

For PXRD measurements the samples were ground and sealed in glass capillaries (0.3 mm outer diameter, Hilgendberg, Malsfeld). After centering the capillary on a rotating goniometer head, data was collected on a Stoe Stadi P diffractometer (STOE & Cie GmbH, Darmstadt) in a modified Debye-Scherrer geometry with Cu-Kα_1 radiation ($\lambda = 1.54060$ Å) or Ag-Kα_1 radiation ($\lambda = 0.55942$ Å) equipped with a MYTHEN 1K strip detector and a Ge(111) monochromator.

The Rietveld refinements for YN, LaN and LuN were carried out on the structural models described in the literature using the program package TOPAS Academic [3, 5–8]. The lattice parameters as well as the occupancy of the N position were freely refined. The peak profiles for all refinements were described with the fundamental parameter approach. The background was modeled by a shifted Chebyshev polynomial [9,10]. The results were plotted using Origin [11]. For LaN and LuN the metal-to-nitrogen ratio is 1:1 by means of PXRD. For YN, the refinement showed a small deviation from the ideal 1:1 stoichiometry, resulting in the sum formula $YN_{0.85}$.

Table S1: Crystallographic data of YN, LaN, and LuN from Rietveld refinement. Standard deviations are given in parenthesis.

**Formula**	$\mathbf{YN_{0.85}}$	**LaN**	**LuN**
Crystal system		cubic	
Space group		$Fm\bar{3}m$ (no. 225)	
Lattice parameters / Å	$a = 4.88483(4)$	$a = 5.29384(5)$	$a = 4.75135(5)$
Cell volume /Å^3	116.560(3)	148.359(4)	107.263(3)
Formula units per unit cell		4	
Density / g·cm^{-3}	5.741	6.846	11.702
Molecular weight / g·mol^{-1}	102.91	152.90	188.96
Diffractometer		STOE Stadi P	
Radiation	Cu-Kα_1 ($\lambda = 1.54059$ Å)	Ag-Kα_1 ($\lambda = 0.55942$ Å)	
Detector		MYTHEN 1K	
Monochromator		Ge(111)	
2θ range / °	$5.0 < 2\theta < 100.47$	$1.0 < 2\theta < 45.205$	$1.0 < 2\theta < 40.180$
Step width / °		0.015	
Data points	6499	2948	2613
Total number of reflections	8	22	12
Refined parameters	14	14	14
Background function		Shifted Chebyshev	
Number of background parameters		6	
Goodness of fit	1.437	1.131	1.391
R_p; R_{wp}	0.048; 0.061	0.070; 0.098	0.056; 0.077
R_{exp}; R_{bragg}	0.043; 0.047	0.0872; 0.017	0.057; 0.026

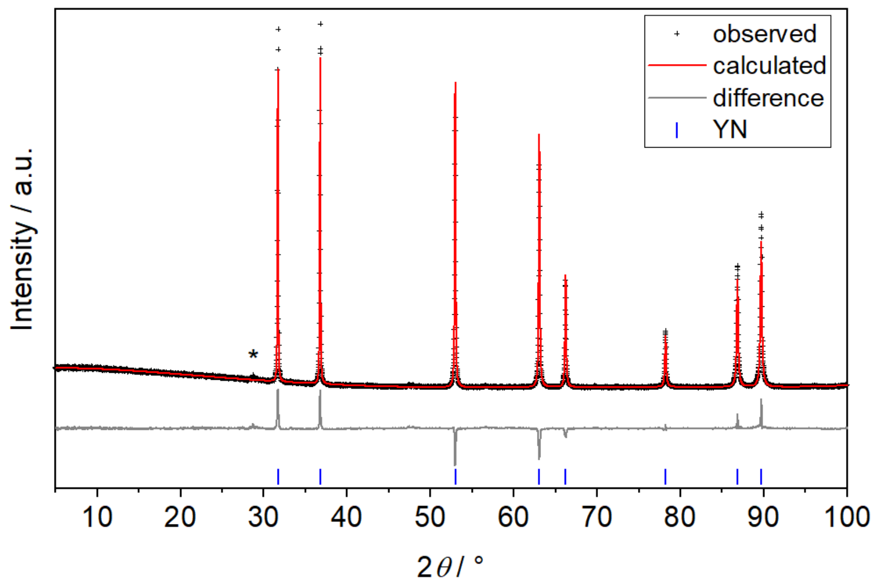

Figure S1: Result of the Rietveld refinement of YN. Observed (black crosses), calculated (red line) powder X-ray diffraction pattern (Cu-Kα_1, $\lambda = 1.54059$ Å) and difference plot (grey line). Position of Bragg reflections of YN are given as blue bars. The ratio of Y to N deviates from the ideal stoichiometry. The N-position is occupied to 84.6(8)%, leading to a sum formula of $YN_{0.85}$. The most intense Bragg reflection of Y_2O_3 is marked with an asterisk but the overall amount of this side phase is too small for a stable refinement.

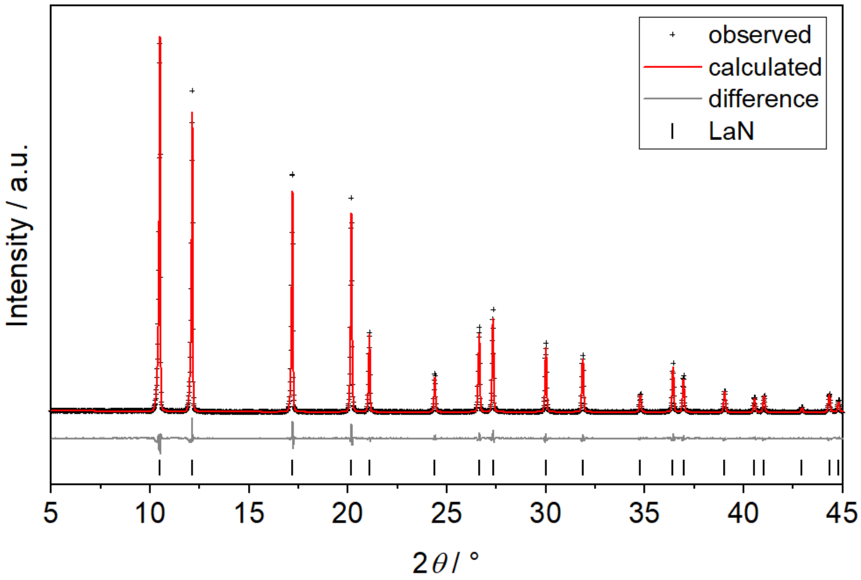

Figure S2: Result of the Rietveld refinement of LaN. Observed (black crosses), calculated (red line) powder X-ray diffraction pattern (Ag-Kα_1, $\lambda = 0.55942$ Å) and difference plot (grey line). Position of Bragg reflections of LaN are given as black bars. The ratio of La to N is 1:1 by means of PXRD.

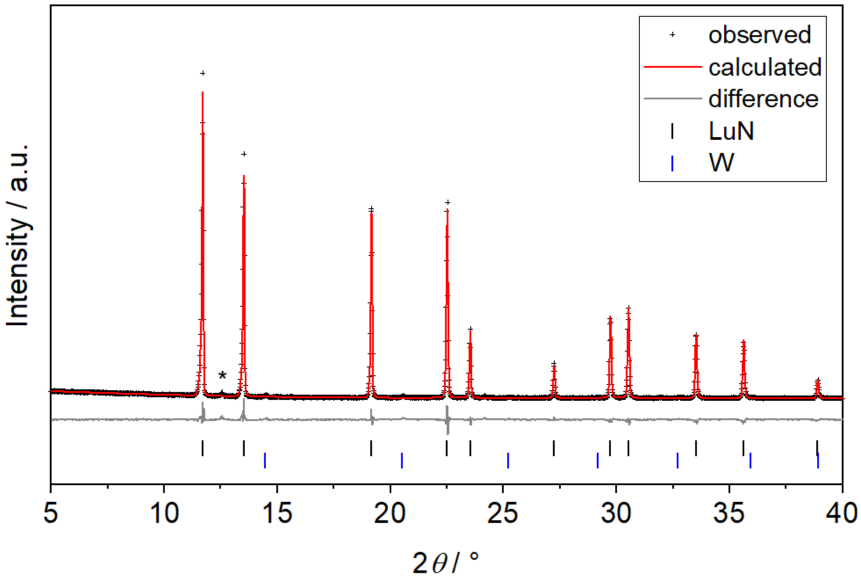

Figure S3: Result of the Rietveld refinement of LuN. Observed (black crosses), calculated (red line) powder X-ray diffraction pattern (Ag-Kα_1, $\lambda = 0.55942$ Å) and difference plot (grey line). Position of Bragg reflections of LuN are given as black bars. The ratio of Lu to N is 1:1 by means of PXRD. Although, the amount of W (crucible material) is too small for a stable refinement, the Bragg reflections are visible. Their positions are marked by blue bars. A Bragg reflection of an unidentified impurity is marked with an asterisk.

3 NMR Spectra Simulations

The NMR spectra in the figures S4 and S5 were both simulated with the Simpson [12] package (version 4.2.1) to compare the calculated sideband intensities with the experimental ones. It should be noted that no influences by the chemical shift interaction has been taken into account and that the simulated spectral lines are much narrower than the experimental ones because the calculation does not reflect any structural imperfections.

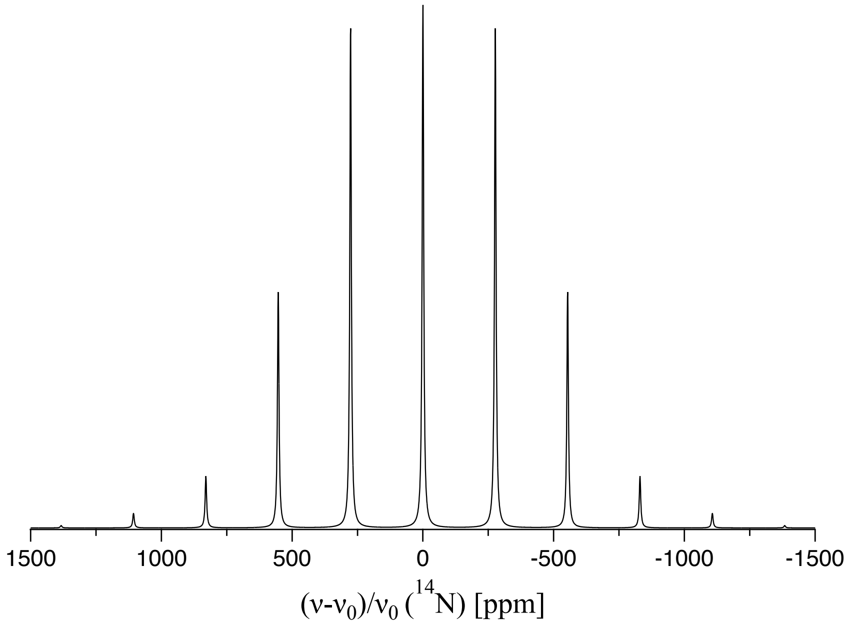

Figure S4: ^{14}N spectrum simulated with only first-order quadrupolar coupling effects and values of $\chi = 43$ kHz and $\eta_Q = 0$.

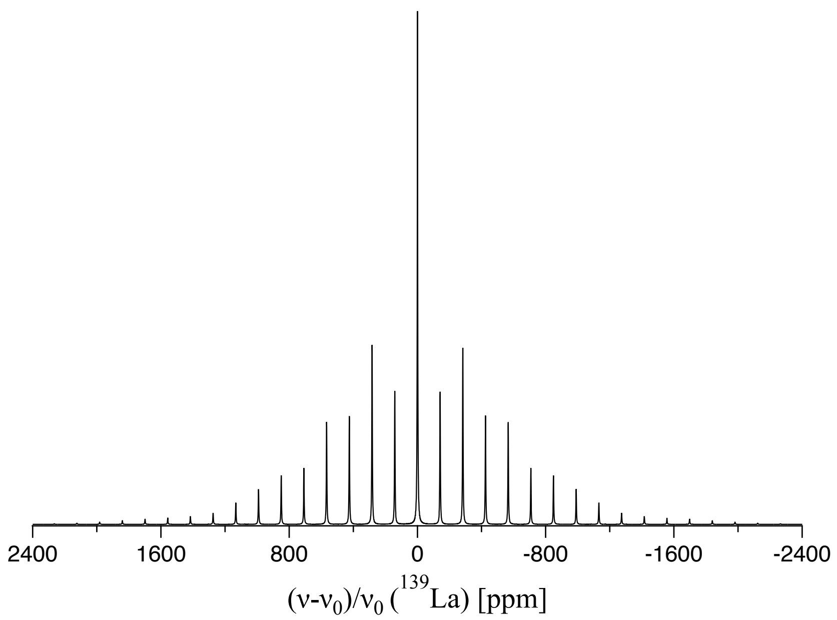

Figure S5: ^{139}La spectrum simulated with first- and second-order quadrupolar coupling effects and values of $\chi = 700$ kHz and $\eta_Q = 0$.

References

[1] Wachter, P. Physical Properties of Some Stoichiometric Rare Earth Nitride Single Crystals. *Adv. Mater. Phys. Chem.* **2015**, *5*, 96–131, https://doi.org/10.4236/ampc.2015.53013.

[2] Schnick, W.; Huppertz, H.; Lauterbach, R. High temperature syntheses of novel nitrido- and oxonitrido-silicates and sialons using rf furnaces. *J. Mater. Chem.* **1999**, *9*, 289–296, https://doi.org/10.1039/A803900C.9.

[3] Rietveld, H.M. A profile refinement method for nuclear and magnetic structures. *J. Appl. Crystallogr.* **1969**, *2*, 65–71, https://doi.org/10.1107/S0021889869006558.

[4] Schütze, M. Feinreinigung von Gasen mit einem hochaktiven Kupferkatalysator. *Angew. Chem.* **1958**, *70*, 697–699, https://doi.org/10.1002/ange.19580702206.

[5] Vendl, A. Über die gegenseitige Mischbarkeit von LaN, CeN, NdN und GdN. *J. Nucl. Mater.* **1979**, *79*, 246–248, https://doi.org/10.1016/0022-3115(79)90452-5.

[6] Holleck, H.; Smailos, E. Mischnitride von Thorium mit Seltenen Erden. *J. Nucl. Mater.* **1980**, *91*, 237–239, https://doi.org/10.1016/0022-3115(80)90054-9.

[7] Klesnar, H.P.; Rogl, P. Phase relations in the ternary systems rare-earth metal(RE) - boron - nitrogen, where RE = Tb, Dy, Ho, Er, Tm, Lu, Sc and Y. *High Temp. – High Pressures* **1990**, *22*, 453–457.

[8] Coelho, A.A. TOPAS-Academic, Version 6, Brisbane, **2016**.

[9] Cheary, R.W.; Coelho, A. A Fundamental Parameters Approach to X-ray Line-Profile Fitting. *J. Appl. Crystallogr.* **1992**, *25*, 109–121, https://doi.org/10.1107/S0021889891010804.

[10] Cheary, R.W.; Coelho, A.A.; Cline, J.P. Fundamental Parameters Line Profile Fitting in Laboratory Diffractometers. *J. Res. Natl. Inst. Stand. Technol.* **2004**, *109*, 1–25, https://doi.org/10.6028/jres.109.002.

[11] OriginPro, Version 2019b, OriginLab Corporation, Northhampton, USA, **2019**.

[12] Bak, M.; Rasmussen, J.T.; Nielsen, N.C. SIMPSON: A general simulation program for solid-state NMR spectroscopy. *J. Magn. Reson.* **2011**, *213*, 366–400, https://doi.org/10.1016/j.jmr.2011.09.008.
